# Transition between bipolar and abnormal bipolar resistive switching in amorphous oxides with a mobility edge

**DOI:** 10.1038/s41598-021-93777-6

**Published:** 2021-07-13

**Authors:** Christiane Ader, Andreas Falkenstein, Manfred Martin

**Affiliations:** 1 Institute of Physical Chemistry, RWTH Aachen University, 52074 Aachen, Germany; 2 JARA-CSD, Forschungszentrum Jülich and RWTH Aachen University, Aachen, Germany; 3 JARA-FIT, Forschungszentrum Jülich and RWTH Aachen University, Aachen, Germany

**Keywords:** Materials science, Theory and computation

## Abstract

Resistive switching is an important phenomenon for future memory devices such as resistance random access memories or neuronal networks. While there are different types of resistive switching, such as filament or interface switching, this work focuses on bulk switching in amorphous, binary oxides. Bulk switching was found experimentally in different oxides, for example in amorphous gallium oxide. The forms of the observed current–voltage curves differ, however, fundamentally. Even within the same material, both abnormal bipolar and normal bipolar resistive switching were found. Here, we use a new drift–diffusion model to theoretically investigate bulk switching in amorphous oxides where the electronic conductivity can be described by Mott’s concept of a mobility edge. We show not only that a strong, non-linear dependence of the electronic conductivity on the oxygen content is necessary for bulk switching but also that changing the geometry of the memristive device causes the transition between abnormal and normal bipolar switching.

## Introduction

Today, there is a rising demand for powerful memory devices, while the current technology has nearly reached its limitations^1,2^. In this context, memristive devices got attention for their use as resistance random access memories (ReRAM) or for neuronal networks. Depending on the preceded voltage, the memristive device can be switched between two states, a high resistive state (HRS) and a low resistive state (LRS)^3^. Several experimental investigations have shown that gallium oxide and especially amorphous, substoichiometric gallium oxide thin films (a-GaO$_n$, $n<1.5$) exhibit resistive switching^4–11^. In contrast to other known resistive switching oxides, such as titania^12,13^ or strontium titanate^14,15^, a-GaO$_n$ does not need a forming process^4,6,8,9^. According to both experimental and simulation results, the resistive switching can be based on a change in bulk resistance due to the migration of oxygen ions between the blocking electrodes of the device^4,6,8,9^. Therefore, the resistance scales with the size of the memristive device, which is an advantage for the downsizing of devices in later applications. For this type of bulk switching, there were two different forms for the *I*–*V* curves reported. The curves can either cross at the point of origin, which is called *bipolar* switching, or they can touch, which is called *abnormal bipolar* switching or sometimes *complementary* resistive switching^16^. Both forms have been found experimentally in gallium oxide, but without a connection between them^4,6,8,9^. Table S1 in the supplementary information summarises these experimental results and shows that there is neither agreement on which mechanism is responsible for switching in gallium oxide nor which switching behaviour is observed at all. Even the theory that an asymmetry in the electrode material is sufficient for normal bipolar resistive switching is not supported by the experimental results in this table^17^. The aim of this work is to shed more light on this problem. Therefore, we concentrate only on bulk switching and do not include any effects connected to the electrodes like interface barriers or electrode materials.

Work already exists on materials which show both unipolar and bipolar behaviour or bipolar behaviour with opposite voltage polarities within the same material^18,19^. However, the results in these studies do not apply to the gallium oxide case, since both rely on filaments in their explanation. In order to understand the observed differences in the *I*–*V* curves for bulk switching of a-GaO$_n$, we performed numerical simulations of the corresponding transport equations. We used a new drift–diffusion model for the movement of oxygen ions that includes Mott’s mobility edge concept for the conductivity of electrons in an amorphous oxide and allows variations of the electrode sizes.

The first model to simulate resistive switching is the famous phenomenological model of Strukov et al.^20^. Due to its 1D nature it cannot consider different electrode sizes. Gale et al. explicitly considered different electrode sizes, but the resulting switching behaviour -normal bipolar or abnormal bipolar—was not investigated^21^. The recent work of Panda et al. is a very comprehensive review on modelling and simulation of resistive random access memories, reviewing more than 200 publications on this topic^22^. The review confirms, that there seems to be no model in literature where the effect of different electrode sizes on the switching type was investigated. The review also shows that the influence of Mott’s concept of a mobility edge for the electronic conductivity in an amorphous oxide has not been considered until now in modelling resistive switching.

Our model as described in “Model” is based on experimental observations and data for amorphous substoichiometric gallium oxide, but is equally applicable to other amorphous oxides. Special focus is on the geometry of the memristive device, especially the sizes of the top electrode (TE) and the bottom electrode (BE). First, we conducted several parameter variations in a one-dimensional geometry and then continued with quasi 3D-simulations with a rotational symmetric model to vary the sizes of the electrodes.

## Model

**Figure 1:**
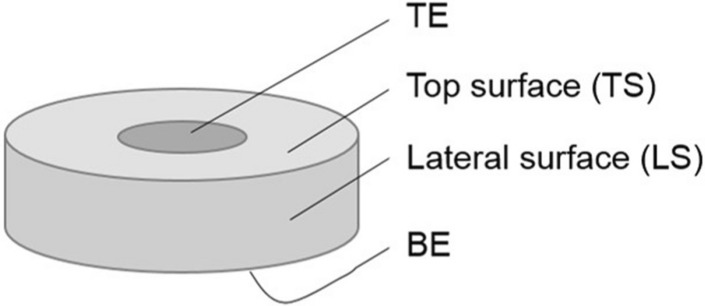
Geometry of the 2D rotational symmetric model. The oxide (light grey) completely covers the bottom electrode (BE), while the top electrode (TE) does not have to cover the whole top of the oxide, but can be smaller as shown here.

In our model, we consider a layer of an amorphous, non-stoichiometric oxide MO$_n$ between two ion-blocking electrodes as shown in Fig. 1.

Since we assume the metal ions to be immobile (${c_{\mathrm{M}^{\mathrm{z}+}}}=\hbox {const}.$) in the oxide, only oxygen ions and electrons remain as two mobile species. However, the same results would be obtained, if we assumed that the oxygen ions were immobile and the metal cations mobile. In addition, we assume that the oxygen ions are only mobile inside the oxide layer and cannot migrate into the electrodes or react with them. There is also no oxygen exchange with the environment. According to findings in gallium oxide, we treat the amorphous highly substoichiometric oxide in our model as electron-rich and regard it as a self-doped semiconductor^23^. Due to the high charge carrier concentration, we can assume charge neutrality in the whole oxide layer. Thus, the electron concentration can be calculated from the location- and time-dependent oxygen ion concentration and the fixed metal ion concentration at any given position *x* and time *t*. Therefore, we choose $c_{\mathrm{O}^{2-}}(x,t)$ as a first function for our model.

Since the applied voltage $V_{\text {ext}}$ between TE and BE corresponds to the difference of the electrochemical potentials of the electrons in the electrodes $\Delta \eta _{e{^-}}$, we choose this potential as a second independent function. Thus, our transport model needs two partial differential equations for the two functions $c_{\mathrm{{O}}^{2-}}(x,t)$ and $\eta _{\mathrm{e}^{-}}(x,t)$.

For the oxygen movement, we use a drift–diffusion model. The flux $j_{\mathrm{{O}}^{2-}}$ of the oxygen ions is given by

1 $$\begin{aligned} j_{\mathrm{{O}}^{2-}} = -\frac{\sigma _{\mathrm{{O}}^{2-}}}{4F^2} \nabla \eta _{\mathrm{{O}}^{2-}} \end{aligned}$$

where $\sigma _{\mathrm{{O}}^{2-}}$ is the oxygen ion conductivity, *F* the Faraday constant, *R* the ideal gas constant, *T* the absolute temperature and $\nabla \eta _{\mathrm{{O}}^{2-}}$ the gradient of the electrochemical potential of the oxygen ions^24,25^. To express $\eta _{\mathrm{{O}}^{2-}}(x,t)$ in terms of the two functions $c_{\mathrm{{O}}^{2-}}(x,t)$ and $\eta _{\mathrm{e}^{-}}(x,t)$, we first consider the equilibrium in Eq. (2). The electrochemical potential of the oxygen ions can therefore be expressed by the chemical potential of oxygen $\mu _{\mathrm{{O}}}$ and the electrochemical potential of the electrons $\eta _{\mathrm{e}^{-}}$ according to Eq. (3).

2 $$\begin{aligned}& {\mathrm{O}^{2-}} \rightleftharpoons {\mathrm{O}} +2{\mathrm{e}}^{-} \end{aligned}$$

3 $$\begin{aligned}&\eta _{\mathrm{{O}}^{2-}} = \mu _{\text {O}} + 2\ \eta _{\mathrm{e}^{-}} \end{aligned}$$

Assuming ideal behaviour for oxygen ions and electrons (simple drift–diffusion model) and considering the charge neutrality (Eq. 4)

4 $$\begin{aligned} z\ c_{{\mathrm{M}^{\mathrm{z}+}}} = 2\ c_{\mathrm{{O}}^{2-}} + c_{\mathrm{e}^{-}} \end{aligned}$$

the driving force for the oxygen ions $\nabla \eta _{\mathrm{{O}}^{2-}}$ can be written as

5 $$\begin{aligned} \nabla \eta _{\mathrm{{O}}^{2-}}&= \gamma \nabla c_{\mathrm{{O}}^{2-}} +2\nabla \eta _{e^-} \end{aligned}$$

whereby $\gamma$ is a thermodynamic factor as shown in Eq. (6).

6 $$\begin{aligned} \gamma = \frac{RT \left( z \ c_{{\mathrm{M}^{\mathrm{z}+}}} + 2 \ c_{\mathrm{{O}}^{2-}} \right) }{c_{\mathrm{{O}}^{2-}} \left( z \ c_{{\mathrm{M}^{\mathrm{z}+}}} - 2 \ c_{\mathrm{{O}}^{2-}} \right) } \end{aligned}$$

Equation (5) shows that the oxygen ions are driven by their local concentration gradient and the local gradient of the electrochemical potential of the electrons, which is due to the applied voltage.

The continuity equation for oxygen ions then leads to the first partial differential equation:

7 $$\begin{aligned} \frac{\partial c_{\mathrm{{O}}^{2-}}}{\partial t} = -\nabla j_{\mathrm{{O}}^{2-}} = \nabla \left( \frac{\sigma _{\mathrm{{O}}^{2-}}}{4F^2} \left( \gamma \nabla c_{\mathrm{{O}}^{2-}} + 2\ \nabla \eta _{\mathrm{e}^{-}} \right) \right) \end{aligned}$$

For this partial differential equation in one dimension, the two following boundary conditions for blocking electrodes, where the BE is at $x=0$ and the TE at a distance $x=d$,

8 $$\begin{aligned}&j_{\mathrm{{O}}^{2-}}(x =0,t)=0 \end{aligned}$$

9 $$\begin{aligned}&j_{\mathrm{{O}}^{2-}}(x =d,t)=0 \end{aligned}$$

and the initial condition of a homogeneous starting distribution of the oxygen ions with a starting concentration of $c_{{\mathrm{{O}}^{2-}}}^{0}$ (Eq. 10) are used.

10 $$\begin{aligned} c_{\mathrm{{O}}^{2-}}({x},t=0)=c_{{\mathrm{{O}}^{2-}}}^{0} \end{aligned}$$

In three dimensions, there are some additional boundaries (see Fig. 1) with new boundary conditions. For all boundaries there is no flux of oxygen ions perpendicular to all surfaces including the top (TS) and lateral surface (LS) (see Fig. 1) as shown in Eq. (11), where $\vec{n}$ is the normal vector of the corresponding surface.

11 $$\begin{aligned} \vec{j}_{\mathrm{{O}}^{2-}} \cdot \vec{n}=0 \end{aligned}$$

The second partial differential equation for $\eta _{\mathrm{e}^{-}}$ is based on the dynamic charge neutrality. The static charge neutrality (4) is valid for any given moment in the whole sample. Thus, the charge density $\rho$

12 $$\begin{aligned}&\rho = F \left( z\ c_{{\mathrm{M}^{\mathrm{z}+}}}-2\ c_{\mathrm{{O}}^{2-}} - c_{\mathrm{e}^{-}}\right) \end{aligned}$$

is always zero. According to the continuity equation for the charge (Eq. 13), the divergence of the total electric current density *i* must be zero, where *i* is given by the sum of electronic and oxygen ion partial current densities (Eq. 14).

13 $$\begin{aligned}&\frac{\partial \rho }{\partial t}=-\nabla i =0 \end{aligned}$$

14 $$\begin{aligned}&i=i_{\mathrm{e}^{-}}+i_{\mathrm{{O}}^{2-}} \end{aligned}$$

If we consider that the electronic flux $j_{\mathrm{e}^{-}}$ is given by

15 $$\begin{aligned}&j_{\mathrm{e}^{-}}=-\frac{\sigma _{\mathrm{e}^{-}}}{F^2}\nabla \eta _{\mathrm{e}^{-}} \end{aligned}$$

we obtain the second partial differential equation (Eq. 16)^24^.

16 $$\begin{aligned}&\nabla \left( \frac{\sigma _{\mathrm{e-}}+\sigma _{\mathrm{{O}}^{2-}}}{F} \nabla \eta _{\mathrm{e-}} + \frac{\sigma _{\mathrm{O}^{2-}}}{2F} \nabla \mu _{\mathrm{O}} \right) =0 \end{aligned}$$

For this partial differential equation the boundary conditions can be derived as follows. The difference of the electrochemical potentials of the electrons between bottom and top electrode can be written as

17 $$\begin{aligned} \Delta \eta _{{\mathrm{e}^-}}(\hbox {TE/BE}) = \Delta \mu _{{\mathrm{e}^-}}(\hbox {TE/BE}) - F \Delta \Phi (\hbox {TE/BE}) \end{aligned}$$

where $\Delta \mu _{{\mathrm{e}^-}}(\hbox {TE/BE})$ is the difference of the chemical potentials of the electrons in the TE and BE and $\Delta \Phi (\hbox {TE/BE})$ the difference of electrostatic potentials. Since bottom and top electrode consist of the same material, $\mu _{{\mathrm{e}^-}}(\hbox {BE})$ and $\mu _{{\mathrm{e}^-}}(\hbox {TE})$ are identical. Assuming electrochemical equilibrium for the electrons at the contact between electrode and oxide, $\eta _{{\mathrm{e}^-}}(\hbox {electrode})=\eta _{{\mathrm{e}^-}}(\hbox {oxide})$, the difference of the electrochemical potentials of electrons within the oxide is given by

18 $$\begin{aligned} \Delta \eta _{{\mathrm{e}^-}}(\hbox {oxide}) = - F \Delta \Phi (\hbox {TE/BE}) = - F V_{\mathrm{ext}} \end{aligned}$$

where $V_{\mathrm{ext}}$ is the external voltage. Using the bottom electrode as the ground electrode, the boundary conditions in one dimension are

19 $$\begin{aligned}&\eta _{\mathrm{e}^{-}}(x = 0,t)=0 \end{aligned}$$

20 $$\begin{aligned}&\eta _{\mathrm{e}^{-}}(x = d,t)= -F V_{\text {ext}}(t) \end{aligned}$$

For the remaining boundaries in three dimensions (TS and LS, see Fig. 1) there is no flux of electrons perpendicular to these surfaces analogous to the boundary conditions for the oxygen ions in Eq. (11).

To obtain both partial differential equations (Eqs. 7, 16 ) only in dependence of $c_{\mathrm{{O}}^{2-}}$ and $\eta _{{\mathrm{e}^-}}$, the conductivities for both mobile species, $\sigma _{\mathrm{{O}}^{2-}}$ and $\sigma _{{\mathrm{e}^-}}$, have to be expressed in dependence of $c_{\mathrm{{O}}^{2-}}$ and $\eta _{\mathrm{e}^{-}}$ as well. For $\sigma _{\mathrm{{O}}^{2-}}$ the Nernst–Einstein equation is used (Eq. 21)^26^.

21 $$\begin{aligned} \sigma _{\mathrm{{O}}^{2-}}= \frac{4 F^2 D_{\mathrm{{O}}^{2-}} c_{\mathrm{{O}}^{2-}}}{R T} \end{aligned}$$

**Figure 2:**
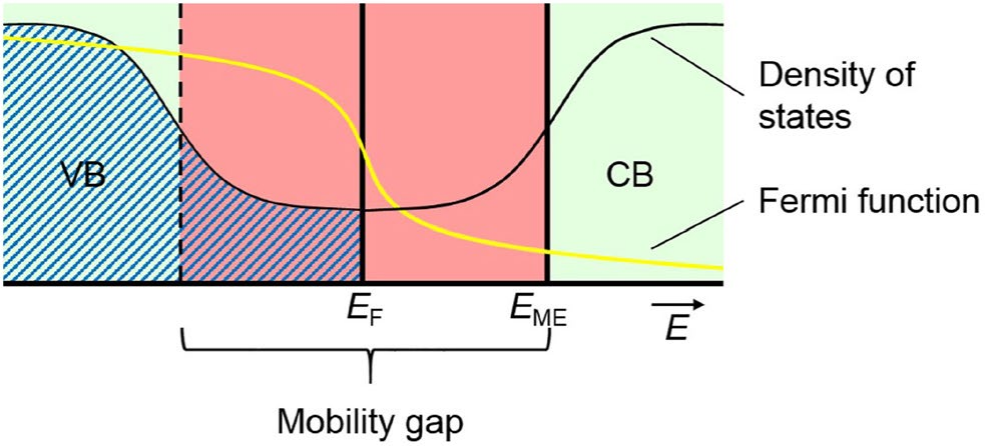
Schematic density of states in amorphous solids. Occupation for energies up to the Fermi energy $E_{{\mathrm{F}}}$ for $T=0\,{\text {K}}$ is shown in blue hatched. Occupations for higher temperatures have to be calculated using the Fermi function (yellow). Within the mobility gap (red area) states are localised. Only electrons above the mobility edge (ME) (right green area) contribute to the electronic conductivity while electrons in the localised states are immobile and therefore have no contribution to the conductivity^27,28^.

The conductivity of the electrons is obtained using a model based on the work of Anderson and Mott^27,28^. In amorphous solids, the valence band (VB) maximum and the conduction band (CB) minimum are not as clearly defined as in crystalline solids (band tailing, see Fig. 2). The states in the band tails are localised, resulting in a mobility gap.

If the Fermi energy lies within the mobility gap, electronic conduction is only possible by thermal excitation of electrons from the Fermi level $E_{{\mathrm{F}}}$ to states above the mobility edge $E_{{\mathrm{ME}}}$. According to Mott, the electronic conductivity can then be expressed by Eq. (22)^28^,

22 $$\begin{aligned} \sigma _{\mathrm{e}^{-}}=\sigma _{{\mathrm{e}^{-}},0} {\text {e}}^{-\frac{E_{\text {ME}}-E_{\text {F}}}{k_{\text {B}}T}} \end{aligned}$$

$\sigma _{{\mathrm{e}^{-}},0}$ is thereby the conductivity at infinitely high temperatures, while $E_{\text {ME}}-E_{\text {F}}$ can be seen as an activation energy for the excitation from the Fermi energy to the mobility edge. With the assumption that the density of states is roughly level in the vicinity of the Fermi energy, the Fermi energy itself is—in a first approximation—linearly dependent on the electron concentration. Assuming a constant mobility edge, the activation energy $E_{\mathrm{ME}}-E_{{\mathrm{F}}}$ (Eq. 22) decreases linearly with increasing electron concentration, $E_{\mathrm{ME}}-E_{{\mathrm{F}}}=r-s\times c_{\mathrm{e}^{-}}$ with two empiric parameters *r* and *s*. Thus, the electronic conductivity depends in this simple model exponentially on the electron concentration (or the oxygen ion concentration, see Eq. 4), in contrast to a crystalline semiconductor, where the conductivity depends linearly on the electron concentration. The parameters $\sigma _{{\mathrm{e}^{-}},0}$, *r* and *s* can be calculated using experimental data of the electronic conductivity as a function of the oxygen content.

We performed the numeric simulations of the transport equations using the finite element method (COMSOL Multiphysics, see “Methods”) first in a one-dimensional geometry and then in a three-dimensional geometry (two-dimensional rotationally symmetric geometry).

## Parametric study

As explained above, we performed the formal analysis for amorphous oxides (MO$_n$) with a mobility gap in general, but used for the parametric study as initial, basic parameters (see Table 1) values from experimental studies of gallium oxide a-GaO$_n$^23,29,30^.

With a triangular voltage sweep for the voltage in Eq. (20), we simulated *I*–*V* curves. We used very small voltages to avoid convergence problems in the simulations and reduce simulation time. “Results and discussion for the basic parameter set” explains in detail why larger voltages are problematic, while in “Large external voltage”, we show how voltages of experimental magnitude can be simulated. In addition, with regard to the diffusion coefficient of the oxygen ions, we have taken the most simple assumption that it is independent of the oxygen concentration and thus constant. In the initial state and with the basic parameters from Table 1, the oxide MO$_n$ is essentially an electronic conductor where the electronic conductivity, $\sigma _{\mathrm{e}^{-}}$, is roughly $10^9$ times larger than the ionic conductivity of the oxygen ions, $\sigma _{\mathrm{{O}}^{2-}}$. However, the electronic conductivity is highly dependent on the stoichiometry (see Eq. 22). Varying *n* between 0.8 and 1.2, the electronic conductivity at $T=298$ K ranges over nearly 14 orders of magnitude.

If not stated otherwise, the basic parameters in Table 1 were used.

**Table 1:** Basic parameters for the simulation.

$D_{\mathrm{{O}}^{2-}}$	1 $\times \ 10^{-18} \,{\mathrm{m}^2\,\hbox {s}^{-1}}$	Oxygen diffusion coefficient
*z*	3	Charge number of cation M$^z+$
$n^0$	1.0	Initial composition of MO$_n$
$\sigma _{{\mathrm{e}^{-}},0}$	$30\,{\hbox {S}\,\hbox {cm}^{-1}}$	Conductivity of the electrons at infinite high temperature
*r*	1 eV	Fitting parameter for the electronic conductivity
*s*	$1.5\times 10 ^{-5}\,{\hbox {eV}\,\hbox {m}^3\,\hbox {mol}^{-1}}$	Fitting parameter for the electronic conductivity
$V_{\mathrm{{m}}}$	$1.5\times 10 ^{-5}\,{\hbox {m}^3\,\hbox {mol}^{-1}}$	Molar volume of the oxide MO_n_
*d*	100 nm	Distance between TE and BE
$V_{\mathrm{{max}}}$	0.02 V	Maximum voltage for the triangular voltage sweep *V* $_{\mathrm{ext}}$ (*t*)
		0 $\rightarrow$ $V_{\mathrm{{max}}}$ $\rightarrow$ 0 $\rightarrow$ $-V_{\mathrm{{max}}}$ $\rightarrow$ 0
$t_{\mathrm{{cycle}}}$	120 s (1D)	Time for one cycle equivalent to a sweeprate of $SR={\frac{0.02\,\hbox {V}}{30\,\hbox {s}}\,=\,}\,$ $6.7 \times 10^{-4}\,\hbox {V}\,{\hbox {s}^{-1}}$
	30 s (3D)	$SR={\frac{0.02\,\hbox {V}}{7.5\,\hbox {s}}\,=\,}\,$ $2.7 \times 10^{-3}$ $\,\hbox {V}\,{\hbox {s}^{-1}}$
*T*	298 K	Temperature

### 1D-simulation

The 1D-model simulates the case where both electrodes have the same size and can be regarded as a basis for the subsequent investigation on the influence of different electrode sizes.

#### Results and discussion for the basic parameter set

**Figure 3:**
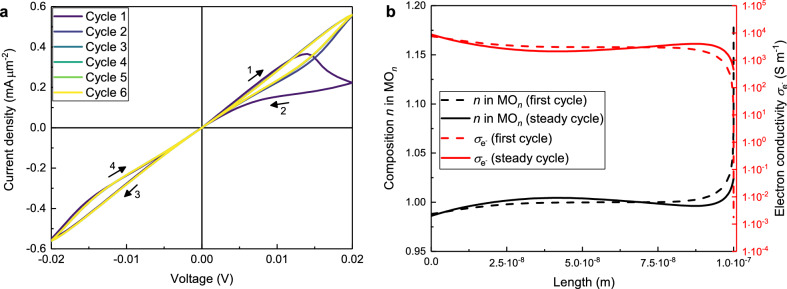
1D-simulation with basic parameters (see Table 1). (**a**) The first six cycles of the *I*–*V* curve are displayed and show abnormal bipolar resistive switching. The numbered arrows indicate the temporal development, which is identical for all cycles. (**b**) Composition profiles (black) and electronic conductivity profiles (note the log-scale for the conductivity in red) after one quarter of a cycle when the maximum voltage is applied. The dotted lines indicate the first cycle while the solid lines indicate the steady cycles. The bottom electrode is on the left side, while the top electrode is on the right side.

The *I*–*V* curve obtained from the numerical solution of the differential Eqs. (7) and (16) subject to the boundary conditions (8)–(10) and (19)–(20) and for the basic parameters in Table 1 is shown in Fig. 3a. In all cycles the device is in the beginning in a LRS and switches within the first quarter cycle into a HRS. Around the point of origin the device changes back into a LRS only to change back after another quarter cycle, resulting in a so called abnormal bipolar behaviour.

Because of the homogeneous starting configuration for the oxygen ions, the first cycle is notably different from the subsequent ones, while after the third cycle a steady *I*–*V* curve is reached, i.e. the following curves are identical. From now on, all *I*–*V* curves are steady *I*–*V* curves.

In order to understand why the device shows resistive switching behaviour at all, a look at Fig. 3b is required. Because of the positive voltage at the TE at the beginning of a cycle, the oxygen ions move towards the ion blocking TE where they accumulate and the oxide becomes polarised (see black curves in Fig. 3b). Charge neutrality is ensured by decreasing the concentration of electrons at the TE. Due to the exponential relationship of electron concentration and electron conductivity (see Eq. 22) the conductivity of the electrons near the TE drops accordingly by orders of magnitude (see red curves in Fig. 3b). Logically at the BE the concentration of the oxygen ions decreases, the concentration of the electrons increases and the conductivity increases as well, but since the dependence is exponential the overall resistance increases and the device switches into the HRS. With increasing concentration of oxygen ions at the TE, the chemical potential of oxygen there increases so that the driving force for the diffusion away from the TE increases as well (see Eq. 7). Directly after reaching its maximum, the voltage is still high enough so that the driving force due to the applied voltage is larger than the driving force due to the chemical potential gradient of oxygen. Therefore, the oxygen ions still move towards the TE. With decreasing voltage in the second quarter of the cycle, the driving force towards the TE decreases as well and the driving force away from the TE prevails. Accordingly, the layer of high oxygen ion concentration at the TE disappears so that the ions are more evenly distributed and the device switches back into the LRS. The second half of a cycle where the positive voltage is at the BE behaves identically. Figure 3 also shows that a larger polarisation (black dotted line in Fig. 3b) leads to a much larger decrease of the conductivity of the electrons (red dotted line in Fig. 3b) and therefore to a larger hysteresis in the *I*–*V* curve (cycle 1 in Fig. 3a) compared to the solid lines and later cycles. In the first cycle at the maximum voltage, the $\sigma _{{\mathrm{e}^-}}$/$\sigma _{\mathrm{{O}}^{2-}}$-ratio directly at the TE is reduced from $10^9$ (initial homogeneous distribution) to $10^3$, while for the later cycles it is only decreased by one order of magnitude to $10^8$. The profiles for the electrochemical potential of the electrons in the supplementary information S1 complete that picture by showing a huge potential drop directly at the TE for the first cycle but a more evenly distributed one over the whole sample for later cycles.

With a higher voltage, the concentration of oxygen ions at the positive electrode is higher as well. When the stoichiometric composition ($n=1.5$) is reached, there are no electrons due to the charge neutrality and the simulation is aborted. In principle that should never happen since the driving force for the diffusion of the oxygen ions away from that electrode is in this case infinitely high (see Eq. 5). In the numerical simulations, though, the layer in front of the electrode with the high concentration of oxygen ions is very thin which leads to numerical problems with the correct calculation of the gradients and therefore no sufficient back diffusion. This problem can be circumvented by using an extremely fine mesh in front of both electrodes, but this comes at a high cost of computational time.

It is important to note that within the simulations resistive switching was only found with the exponential dependence of electronic conductivity on electron concentration (see Eq. 22). With a linear dependence, describing the electronic conductivity in a (crystalline) oxide without a mobility edge, we observed no resistive switching at all (see supplementary information S1.

**Figure 4:**
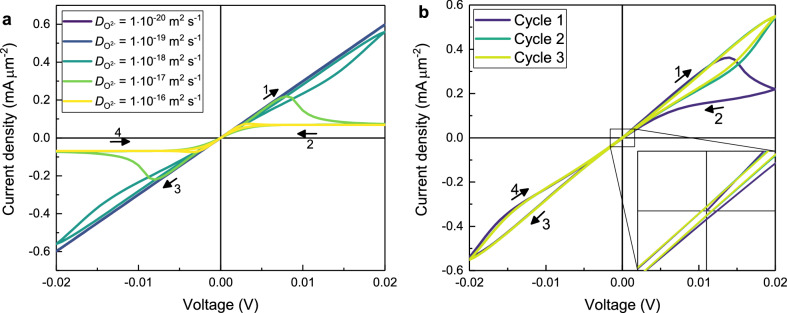
I–V curves for the variation of the diffusion coefficient $D_{\mathrm{{O}}^{2-}}$ in 1D with all other parameters fromTable 1. (**a**) Variation of the diffusion coefficient over five orders of magnitude. (**b**) Simulation with a high diffusion coefficient of the oxygen ions $D_{\mathrm{{O}}^{2-}}=1\times 10^{-10}\,{\text{m}^2\; \text{s}^{-1}}$ and a small cycle time $t_{\text{{cycle}}}= 1.2\times 10^{-6}\, {\text{ s}}.$

**Figure 5:**
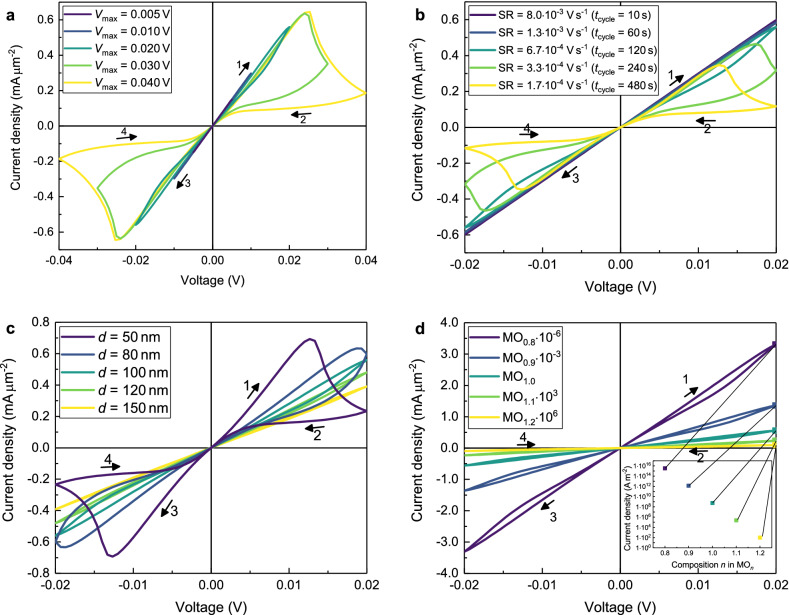
I–V curves for 1D-simulations with parameters from Table 1. (**a**) Variation of the maximum voltage $V_{\text{{max}}}$. (**b**) Variation of the sweeprate SR. (**c**) Variation of the sample’s thickness *d*. (**d**) Variation of the initial composition $n^0$. In order to show all graphs in the same window they are scaled by the factor stated in the legend. The inset shows the unscaled current densities at the voltage of 0.02 V.

For the subsequent parametric study, the diffusion coefficient of the oxygen ions $D_{\mathrm{{O}}^{2-}}$, the maximum voltage $V_{{\max}}$, the sweep rate *SR*, the sample’s thickness *d* and the initial composition of the oxide $n^0$ are examined in the previously explained one-dimensional geometry. All resulting diagrams are shown in Figs. 4 and 5.

#### Variation of the diffusion coefficient of the oxygen ions $D_{\mathrm{{O}}^{2-}}$

Here we take the oxygen diffusion coefficient in Table 1 as a reference. With a smaller diffusion coefficient (purple and blue curves in Fig. 4a), the oxygen ions move slower, so that the polarisation in the sample is small which results in ohmic *I*–*V* curves. The higher the value for the diffusion coefficient is (green to yellow curves (Fig. 4a), the faster the device switches into the HRS, as is expected and can be seen with the yellow curve where the device is already in the HRS at a voltage of 0.005 V. In summary, the existence of a noticeable hysteresis in the *I*–*V* curve at a fixed *SR* is very sensitive to the magnitude of the diffusion coefficient, which is in agreement with an earlier report by Kalaev et al.^31^.

Figure 4b shows the *I*–*V* curve for a simulation with a very high diffusion coefficient of $D_{\mathrm{{O}}^{2-}}\,=\, 1\times 10^{-10}\,{\hbox {m}^2\,\hbox {s}^{-1}}$ and a reduced time for one cycle of $t_{\mathrm{{cycle}}}\,=\, 1.2\times 10^{-6}$ s. The shape is nearly identical to the one with basic parameters in Fig. 3a, because the product of diffusion coefficient and cycle time ($D_{\mathrm{{O}}^{2-}} \times t_{\mathrm{{cycle}}}\,=\, 1.2\times 10^{-16}$ m$^2$) is identical. The only difference is in the high oxygen ion mobility which is now similar to the electronic one. The inset in Fig. 4b shows clearly that the *I*–*V* curve starts in the point of origin, while in all subsequent cycles the *I*–*V* curve crosses the *I*-axis and the *V*-axis outside of the origin. The voltage corresponding to zero current is called electromotive force (*EMF*) and can be calculated by Eq. (23)^32^.

23 $$\begin{aligned} EMF=\frac{1}{2F}\int _0^d\frac{\sigma _{\mathrm{{O}}^{2-}}(x)}{\sigma _{\mathrm{{O}}^{2-}}(x)+ \sigma _{\mathrm{e}^{-}}(x)} \nabla \mu _{\mathrm{{O}}}(x)\, {\mathrm {d}}x \end{aligned}$$

With the parameters from Fig. 4b, Eq. (23) leads to a value of $EMF=\pm 1.88\times 10^{-4}\,\hbox {V}$ which is the same value that can be obtained from the third cycle in Fig. 4b. This phenomenon is also known as nanobattery effect and was already found experimentally for the valence change memory type cells Ti/SrTiO_3_/Pt and Ta/Ta_2_O5_5_/Pt^33^.

#### Variation of the maximum voltage $V_{{\max}}$

Varying the maximum voltage while keeping the sweeprate constant leads to the *I*–*V* curves in Fig. 5a. With a small voltage (purple and blue curves), there is no significant polarisation so that no switching is observed. A larger maximum voltage (green and yellow curve) leads to a large hysteresis between the HRS and the LRS as is needed for applications.

#### Variation of the sweeprate *SR*

In Fig. 5b, the *I*–*V* curves for the variation of the sweeprate are shown. The smaller the sweeprate is, the longer is the time for the oxygen ions to move towards the corresponding electrode and thereby switch the device from HRS to LRS or vice versa. In case of high sweeprates (purple and blue curves) there is again no significant polarisation resulting in ohmic behaviour. Our results are in agreement with the results from Kalaev et al.^34^.

#### Variation of the sample’s thickness *d*

The *I*–*V* curves for the variation of the sample’s thickness are shown in Fig. 5c. For thick samples (yellow and green curve) the oxygen ions can only cover a certain percentage of the distance between the electrodes before the polarity changes which leads to ohmic behaviour. For thinner samples (blue and purple curve) the ions reach the electrode earlier and can accumulate there. In this case, the *I*–*V* curves show more pronounced switching behaviour. Additionally, the sample’s thickness influences the gradient of the electrochemical potential of the electrons $\nabla \eta _{{\mathrm{e}^-}}$. In case of the thickest sample (yellow curve) it is lowest, resulting in a low maximum current density. For the thinnest sample (purple curve) $\nabla \eta _{{\mathrm{e}^-}}$ is much higher, and therefore the driving force for the drift of the oxygen ions is higher as well, which leads to a higher maximum current density.

#### Variation of the initial composition factor $n^0$ in MO$_n$

The influence of the initial stoichiometry is shown in Fig. 5d. The shape of the *I*–*V* curve is the same for all compositions, but the slope depends strongly on the oxygen content and the connected concentration of electrons. The inset in Fig. 5d shows the current density at the maximum voltage of 0.02 V in dependence of the composition. The linearity in that logarithmic plot reflects directly the exponential dependence of the electronic conductivity on the concentration of the electrons in Eq. (22).

#### Large external voltage

**Figure 6:**
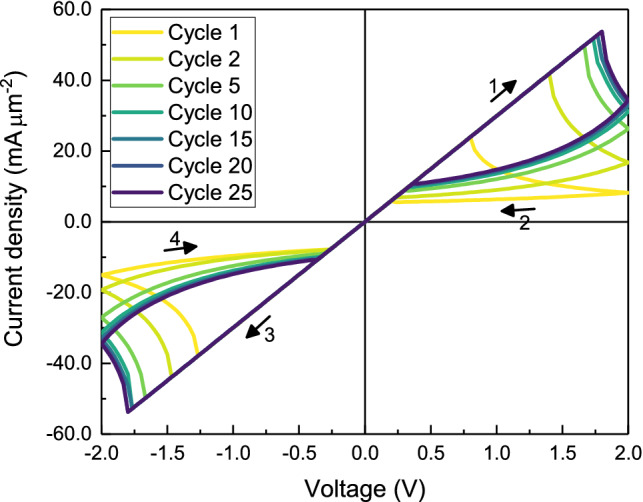
I–V curve for a 1D-simulation with high voltage. The diffusion coefficient for the oxygen ions is $D_{{\mathrm{{O}}^{2-}}}= 1\times 10^{-21}{\text { m}^2{\text{ s}}^{-1}}$ and the voltage is $V_{\mathrm{{max}}}= 2{\text{ V}}.$All other parameters are listed in Table 1.

Before we moved to quasi three-dimensional simulations, we performed additional 1D simulations to make sure that they also work with lower diffusion coefficients and higher voltages. With a diffusion coefficient for the oxygen ions of $D_{\mathrm{{O}}^{2-}}\,=\, 1\times 10^{-21}\,{\hbox {m}^2\,\hbox {s}^{-1}}$ which is in good agreement with experimental values for amorphous gallium oxide^35^ and a voltage of $V_{\mathrm{{max}}}\,=\, 2$ V which is a typical value for experimental studies^6^ we still observe pronounced abnormal bipolar resistive switching. The corresponding current–voltage curve is shown in Fig. 6. Due to the lower diffusion coefficient, the oxygen ions need more time to drift through the sample in order to form the nonhomogeneous starting configuration for the steady *I*–*V* curve which is only reached after about 20 cycles.

### 3D-simulation

In a three-dimensional model, the variation of the TE-area to BE-area ratio is now possible. We performed the simulations in a 2D rotationally symmetric model, which is quasi 3D, in order to save computational time.

**Figure 7:**
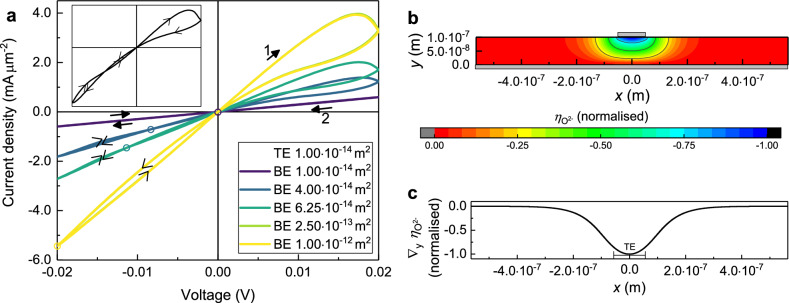
3D-simulations with parameters from Table 1. (**a**) Variation of the size of the bottom electrode (BE). The size of the top electrode (TE) is kept constant, while the diameter of the BE and the oxide layer vary in size. The circles indicate the location of the intersection point. Note that the green curve (${\text {BE}}=2.50\times 10^{-13}\,{\text {m}}^2$) is completely covered by the yellow curve. (**b**) Contour plot of the electrochemical potential of the oxygen ions at the maximum voltage of 0.02 V after the first quarter of a steady cycle. Shown is the cross section across the centre of the device. The electrodes are shown in grey with the TE being much smaller than the BE, corresponding to the yellow curve with a BE-area of $1.00\times 10^{-12}\,\mathrm {m}^2$ in (**a**). (**c**) *y*-component of the gradient of the electrochemical potential of the oxygen ions in (**b**) at the BE ($y=0$).

Figure 7a shows the *I*–*V* curves for the variation of the size of the bottom electrode while the size of the top electrode is constant.

The purple curve where the BE is as large as the TE shows abnormal bipolar switching. As expected by symmetry, this curve is identical to the 1D *I*–*V* curve (see supplementary information S1). For slightly larger bottom electrodes (blue curves) the shape of the *I*–*V* curve changes. Now, there is an intersection point in the third quadrant, for the dark blue curve (${\text {BE}}=4.00\times 10^{-14}\,{\text {m}}^2$) close to the point of origin and for the lighter blue curve (${\text {BE}}=6.25\times 10^{-14}\,{\text {m}}^2$) at around − 0.01 V. For the *I*–*V* curves with even larger bottom electrodes (green and yellow curves) the switching behaviour has changed to normal counter-8 bipolar switching. Both the abnormal bipolar behaviour^8^ and the normal bipolar behaviour^4,6^ have been found experimentally, but without any connection between them. Here, the transition from abnormal to normal bipolar switching can be observed through the movement of the new intersection point by increasing the size of the BE. In case of the abnormal bipolar behaviour, the intersection point is directly in the point of origin while in case of the normal bipolar behaviour it has moved to the most negative voltage. Figure 7a also shows that a critical size for the BE exists after which an increase of the BE does not change the switching behaviour any further (see light green (${\text {BE}}=2.50\times 10^{-13}\,{\text {m}}^2$) and yellow (${\text {BE}}=1.00\times 10^{-12}\,{\text {m}}^2$) curve in Fig. 7a). This critical size of the BE for the given thickness of the sample and the given size of the TE is between $6.25\times 10^{-14}\,{\text {m}}^2$ and $2.50\times 10^{-13}\,{\text {m}}^2$. A reason for this phenomenon can be found in the driving force $\nabla \eta _{\mathrm{{O}}^{2-}}$ for the movement of the oxygen ions. Figure 7b shows the contour plot of the electrochemical potential of oxygen ions at the end of the first quarter of a cycle with the maximum voltage of 0.02 V in a cross section passing through the centre of the device. Its gradient is not perpendicular to the electrodes, but spreads out radially from the TE. Therefore, the oxygen ions do not move only along the shortest path between the electrodes, but also take longer paths along the gradient. Consequently, the device cannot switch nearly instantly back into the LRS when the cycle reaches the point of origin at the end of the second quarter of a cycle. In contrast to the device with equally sized electrodes it remains at the beginning of the third quarter in the HRS. The larger the BE is, the longer it takes for the device to switch back into the LRS which happens exactly at the intersection point. It is also visible that the electrochemical potential of the oxygen ions only changes substantially in the vicinity of the TE. This is even more visible in Fig. 7c where its gradient in *y*-direction is shown along the BE. Directly below the TE the gradient is strong, but further outwards its value approaches zero. Therefore, increasing the BE does not change the switching behaviour further after the critical size of the BE is reached.

The longer pathways for the oxygen ions are also the reason for the adjustment of the sweeprate compared to the sweeprate in the 1D simulations. In order to obtain normal bipolar switching for the *I*–*V* curve with the largest BE (yellow curve in Fig. 7a) the sweeprate has to be increased. If the sweeprate is not large enough, increasing the size of the BE moves the intersection point to smaller voltages, but the critical size for the BE after which the *I*–*V* curve changes no longer is reached before the intersection point has reached the most negative voltage (not shown).

**Figure 8:**
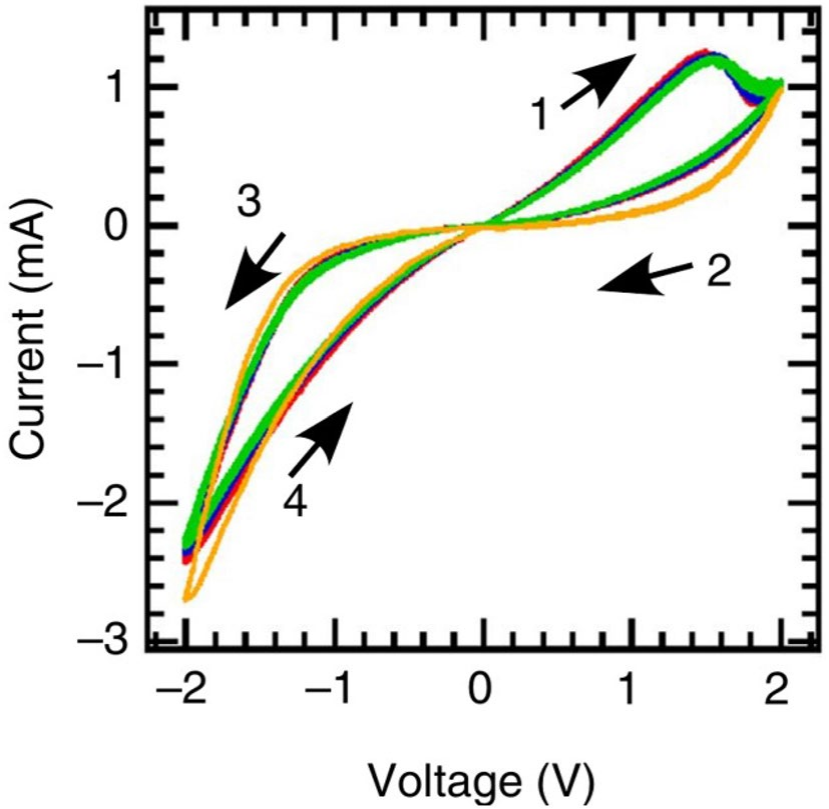
Experimental I–V curve at 25 $^\circ$C of a 90 nm thick substoichiometric amorphous Gallium oxide thin film with a sweeprate of $0.05\,\hbox {V}\,{\hbox {s}^{-1}}$. The TE has a diameter of 200 µm and the BE a size of 1 cm $\times$ 1 cm. Reprinted by permission from Springer Nature Limited: Springer Nature, *Nature Communications*^6^ (Aoki et al). Copyright (2014).

**Figure 9:**
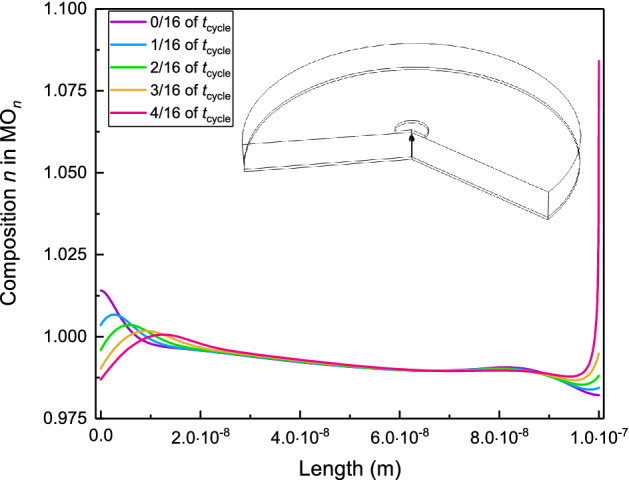
Concentration profiles of the oxygen ions along the rotational axis of the device for a 2D rotational symmetric simulation with basic parameters (see Table 1) and a BE with $(1.0\times 10^{-6}\,\hbox {m})^2$. The BE is at 0 m while the TE is at $1\times 10^{-7}$ m. This is only the first quarter of a steady *I*–*V* curve.

The *I*–*V* curve with the smallest TE/BE ratio in Fig. 7a is now compared to the experimental *I*–*V* curve for a-GaO$_n$ with an even smaller TE/BE ratio in Fig. 8^6^. Besides the s-formed shape of the experimental curve which is probably caused by non-linear contact resistances at the electrodes (e.g. Schottky barriers), both experimental and simulated *I*–*V* curves match qualitatively very well. Especially the characteristic shape in the first quadrant is reproduced and further investigated. Therefore, the different composition profiles of the oxide at different times within the first quarter of a steady *I*–*V* curve are shown in Fig. 9. Starting with a positive voltage at the TE, the oxygen ions move towards it. Therefore, the resistance increases at the TE while it decreases at the BE. Both effects balance each other out which then leads to a slightly bent, but mostly straight *I*–*V* curve before the maximum of the current density in the first quarter of the cycle (see yellow curve in Fig. 7a). Between three sixteenth and four sixteenth of the time for one cycle the oxygen concentration at the BE continues to decrease slowly while the oxygen concentration at the TE suddenly increases strongly. As a consequence, the overall resistance of the device increased and the characteristic maximum of the current density in the first quadrant is formed.

## Conclusions

In this work, we used a new drift–diffusion model to simulate the bulk switching in binary, highly non-stoichiometric, amorphous oxides with a mobility edge for the electronic conductivity. In a one dimensional parametric study, we tested the influence of several parameters such as the diffusion coefficient of oxygen ions, the maximum voltage, the sweeprate, the sample’s thickness and the initial composition of the oxide. We found that the incorporation of Mott’s concept of a mobility edge is essential. Without the exponential dependence of the electronic conductivity on the oxygen concentration no switching was found. By using a high diffusion coefficient and a small cycle time, we were also able to simulate the experimentally found electromotive force of a polarised cell where the *I*–*V* curve does not cross the origin. In a next step, we used a 2D rotational symmetric model to simulate three dimensional devices. The *I*–*V* curves with equal sized electrodes showed an abnormal bipolar behaviour identical to the one–dimensional *I*–*V* curves. By increasing the size of the bottom electrode while keeping the size of the top electrode constant, we observed a transition from abnormal to normal bipolar switching. First, an intersection point appeared near the point of origin, which then moved with increasing size of the bottom electrode to the most negative voltage, leading to normal bipolar switching behaviour. After a critical size of the bottom electrode is exceeded, no further change is observed. This phenomenon is caused by the gradient of the electrochemical potential of the oxygen ions, which is the driving force for the movement of the oxygen ions. Outside the centre of the device, the gradient is not perpendicular to the electrodes, which results in longer diffusion pathways for the oxygen ions and thus longer transition times for the switching between LRS and HRS. Our model can reproduce clearly the normal bipolar switching behaviour that was found experimentally for a-GaO$_n$ ($n\approx$1) (see Fig. 8) where a small ratio of TE-area and BE-area was used. In particular, the comparison of our 3D simulations with experimental results shows good qualitative agreement where the shape of the curve is reproduced very well and explained by the composition profiles. Therefore our model could contribute to the development of memristive switching devices, especially when it comes to finding suitable parameters such as voltage and sweeprate or the best geometry. Finally, we emphasise that our drift diffusion model including Mott’s concept of a mobility edge could be applied to various transport phenomena in amorphous mixed ionic electronic conductors (MIEC).

## Methods

For all simulations, we used version 5.3 of the *Comsol Multiphysics* program without any optional modules.

For the mesh in one dimension, we divided the length into three intervals in order to increase mesh point density at the electrodes. In the one percent next to the electrodes, there are 500 mesh points with Comsol’s *element ratio* of 100, while in the remaining interval, there are 100 mesh points with an *element ratio* of 50 and a symmetric distribution. An exception is the mesh for the 2 V simulations, where we had to use an even finer mesh due to the steep gradients at the electrodes. There, we divided the length into eleven intervals with new intervals beginning after $1\times 10^{-8}$, $1 \times 10^{-7}$, $1 \times 10^{-6}$, $1 \times 10^{-2}$, 5, $100-5$, $100-1 \times 10^{-2}$, $100-1 \times 10^{-6}$, $100-1 \times 10^{-7}$, and $100-1 \times 10^{-8}$ percent of the sample length. In the first and last five intervals, we used 50 mesh points with an *element ratio* of 10 and in the remaining interval 50 mesh points with an *element ratio* of 5 and a symmetric distribution. For both meshes the maximum element size was 2 $\times 10^{-10}$ m, the maximum element growth rate 1.1 and the resolution of narrow regions 1.

For the three-dimensional simulations we used 1000 mesh points at the TE and depending on the size of the BE 1000–10,000 mesh points for the BE with an *element ratio* up to 800. The element size was between 1.1$\times 10^{-8}$ and 2.2$\times 10^{-8}$ m, the maximum element growth rate 1.1, the curvature factor 0.2 and the resolution of narrow regions 1.

For all simulations, we used a timestepping between 0.01 and 0.25 s.

For the one dimensional simulations, we used the PARDISO solver and for the three-dimensional simulations the MUMPS solver. For all models, we set the shape function for the partial differential equation for the concentration of the oxygen ions to *divergence* with a linear element order. For the partial differential equation for the electrochemical potential of the electrons, we used a *lagrange* shape function with quadratic order.

## Supplementary Information

Supplementary Information 1.
