# Supplementary material for: Transition between bipolar and abnormal bipolar resistive switching in amorphous oxides with a mobility edge
Source: Sci Rep. 2021 Jul 13;11:14384. doi: 10.1038/s41598-021-93777-6 (PMC8277833; doi:10.1038/s41598-021-93777-6)
Supplement: Supplementary file 1 — Supplementary Information 1. [file 41598_2021_93777_MOESM1_ESM.pdf]

Supplementary Information

Transition Between Normal and Abnormal Bipolar Resistive Switching in Amorphous Oxides with a Mobility Edge

Christiane Ader[1], Andreas Falkenstein[1], and Manfred Martin[1,2,3,*]

[1]Institute of Physical Chemistry, RWTH Aachen University, 52074 Aachen, Germany
[2]JARA-CSD, Forschungszentrum Jülich and RWTH Aachen University, Germany
[3]JARA-FIT, Forschungszentrum Jülich and RWTH Aachen University, Germany
[*]martin@rwth-aachen.de

Table S1: Overview over experimental work for gallium oxide.

Observed switching behaviour	Proposed mechanism	Same material for both electrodes	Reference
counter 8-figure normal bipolar	interface switching	no	Gao et al. (2010)[1]
8-figure normal bipolar	bulk switching	no	Gao et al. (2010)[1]
8-figure normal bipolar	filament switching	no	Yang et al. (2013)[2]
counter 8-figure normal bipolar	bulk switching	no	Aoki et al. (2014)[3]
unipolar	filament switching	yes	Guo et al. (2015)[4]
abnormal bipolar	interface switching with bulk effects	yes	Guo et al. (2015)[5]
abnormal bipolar	bulk switching with interface effects	yes	Guo et al. (2017)[6]
unipolar	filament switching	no	Zhang et al. (2019)[7]
8-figure normal bipolar	interface switching	no	Gutierrez et al. (2020)[8]

Table S1 shows that there is neither agreement on which mechanism is responsible for switching in gallium oxide nor which switching behaviour is observed at all. Even the theory that an asymmetry in the electrode material is sufficient for normal bipolar resistive switching is not supported by the experimental results in table S1.[9]

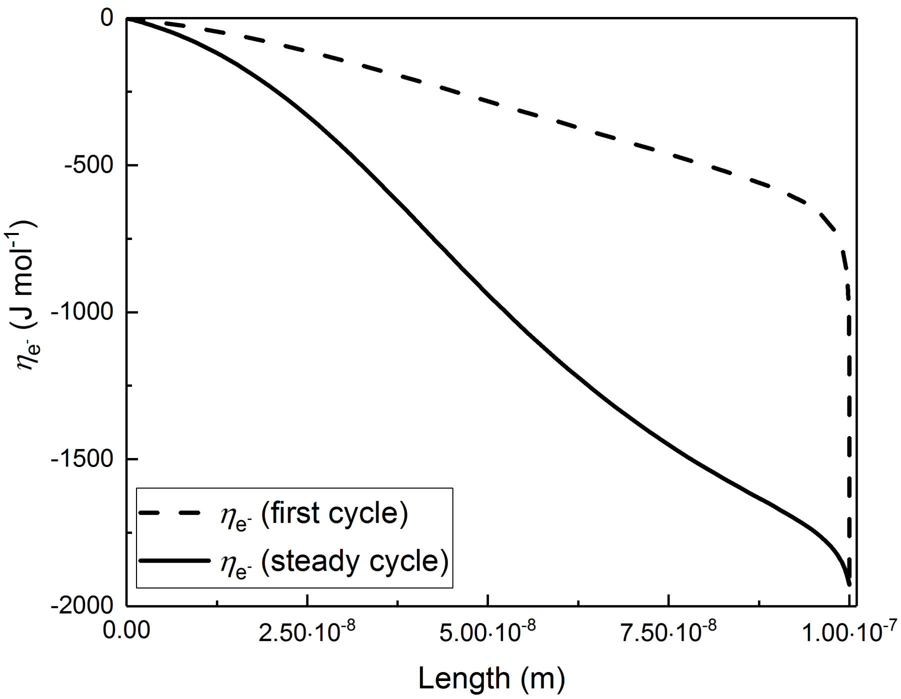

Figure S1: Profile of the electrochemical potential of the electrons after one quarter of a cycle. The dotted line indicates the first cycle with a huge drop of the electrochemical potential of electrons directly at the TE while the solid line indicates the steady cycle where the potential drop is more evenly distributed over the whole sample. This 1D simulation was performed with the basic parameters from table 1).

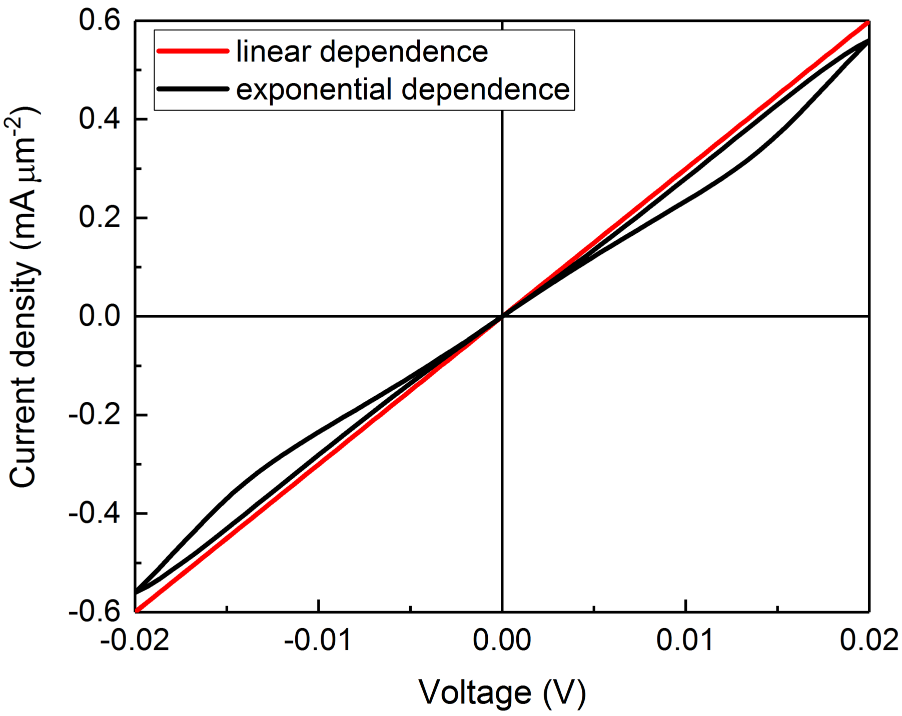

Figure S2: Comparison of the *I-V* curves with a linear and an exponential dependence of the electronic conductivity on electron concentration in 1D. The parameters of the linear dependence are chosen in a way that the electronic conductivities for both simulations are identical for the starting composition. Else the parameters from table 1 are used. The black *I-V* curve shows abnormal bipolar behaviour while the red *I-V* curve shows no resistive switching.

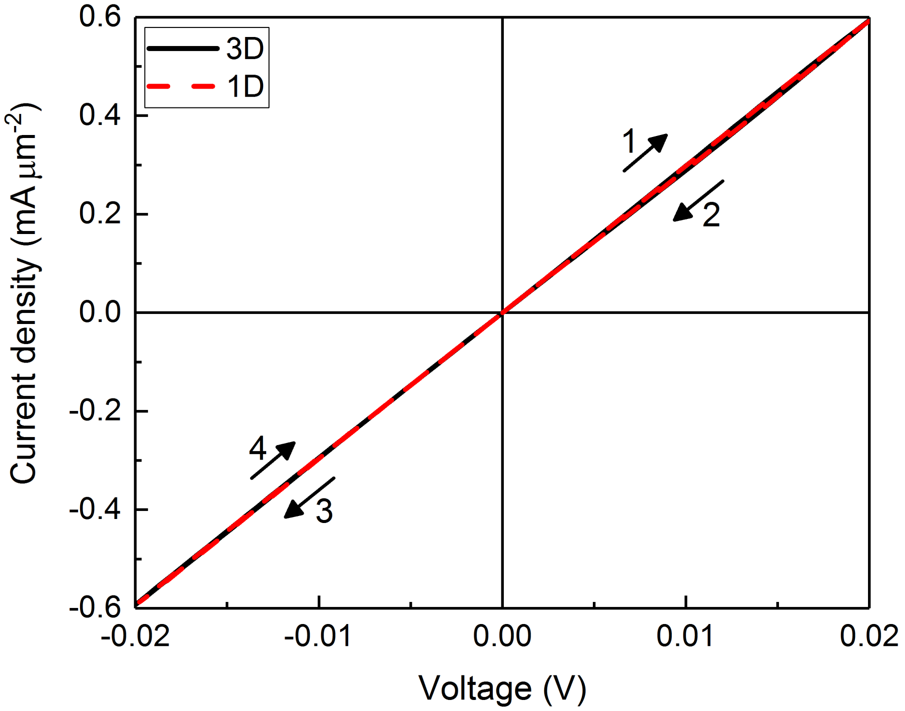

Figure S3: Comparison of the *I-V* curves for 1D and 3D simulations. Both curves are simulated with the 3D parameters from table 1 and equally sized electrodes for the 3D simulation.

References

1. Gao, X. *et al.* Effect of top electrode materials on bipolar resistive switching behavior of gallium oxide films. *Appl. Phys. Lett.* **97**, 193501 (2010).
2. Yang, J.-B. *et al.* Resistive switching characteristics of gallium oxide for nonvolatile memory application. *Thin Solid Films* **529**, 200–204 (2013).
3. Aoki, Y. *et al.* Bulk mixed ion electron conduction in amorphous gallium oxide causes memristive behaviour. *Nat. Commun.* **5**, 3473–3481 (2014).
4. Guo, D. *et al.* Unipolar resistive switching behavior of amorphous gallium oxide thin films for nonvolatile memory applications. *Appl. Phys. Lett.* **106**, 042105 (2015).
5. Guo, D. Y. *et al.* Abnormal bipolar resistive switching behavior in a Pt/$GaO_{1.3}$/Pt structure. *Appl. Phys. Lett.* **107**, 032104 (2015).
6. Guo, D. Y. *et al.* Evidence for the bias-driven migration of oxygen vacancies in amorphous non-stoichiometric gallium oxide. *AIP Advances* **7**, 065312 (2017).
7. Zhang, L., Yu, H., Xiong, L., Zhu, W. & Wang, L. The modification of ultraviolet illumination to resistive switching behaviors in ga_2o_3 memory device. *Journal of Materials Science: Materials in Electronics* **30**, 8629–8635 (2019).
8. Gutiérrez, D., De Sousa, J. A., Mas-Torrent, M. & Crivillers, N. Resistive switching observation in a gallium-based liquid metal/graphene junction. *ACS Appl. Electron. Mater.* **2**, 3093–3099 (2020).
9. Kalaev, D. & Riess, I. On conditions leading to crossing of i–v curve in metal1— mixed-ionic–electronic-conductor— metal2 devices. *Solid State Ionics* **241**, 17–24 (2013).
